# Identification and Multigene Phylogenetic Analysis Reveal *Alternaria* as the Primary Pathogen Causing European Plum (*Prunus domestica*) Brown Spot in Xinjiang, China

**DOI:** 10.3390/jof12010069

**Published:** 2026-01-15

**Authors:** Shuaishuai Sha, Qiuyan Han, Hongyue Li, Wenwen Gao, Jiyuan Ma, Lingkai Xu, Canpeng Fu, Pan Xie

**Affiliations:** 1 School of Advanced Agricultural Sciences, Kashi University, Kashi 844000, China; sha_ksu@sina.com (S.S.);; 2 Xinjiang Zhongyuan Agricultural Science and Technology Co., Ltd., Kashi 844000, China

**Keywords:** European plum, brown spot disease, *Alternaria*, pathogen identification, multigene phylogeny, Xinjiang

## Abstract

European plum (*Prunus domestica*) orchards in the Kashi region, Xinjiang, China, suffer from fruit brown spot disease. The disease typically appears as red spots on the fruit surface that expand into brown necrotic lesions; affected fruit flesh can shrink, and fruits can harden and drop. We isolate and identify pathogens associated with this disease in this plum from five Kashi counties. Of 210 fungal isolates obtained through standard tissue isolation, *Alternaria* accounted for 84.8%, with the remainder comprising species of *Aspergillus* (9.5%), *Diplodia* (3.3%), and *Neoscytalidium* (2.4%). Using PCR amplification and sequencing of five loci, pathogens were identified using multi-gene phylogenetic analyses, combined with observations of colony and spore morphology. Multi-locus sequences of *Alternaria* isolates were highly homologous to those of the *Alternaria alternata* type strain, and we refer them to an *A. alternata* species complex. Pathogenicity tests confirm that *Alternaria* isolates reproduce brown spot symptoms on European plum fruits. By demonstrating that *Alternaria* is the primary pathogen causing brown spot disease in European plum in Xinjiang, we clarify both the fungal species composition and taxonomic placement of the dominant pathogen associated with this disease.

## 1. Introduction

European plum (*Prunus domestica*), hereinafter “plum,” is a major stone fruit grown in temperate regions worldwide. Consumed both fresh and when processed into dried products, this plum holds a unique position in the international fruit market [1]. Food and Agriculture Organization of the United Nations (FAOSTAT) data reveal that China has long ranked first in the world in terms of *Prunus* fruit tree production. Serbia, Romania, Chile, and Italy are also important plum-producing countries [1,2]. In China, Xinjiang is an ideal region for plum production because of its arid climate, minimal rainfall, and considerable day–night temperature differences. Here, Jiashi county, Kashi prefecture, has developed into a large-scale planting base. Public reports indicate that this county now has 570,000 mu of plums; output exceeded 370,000 t in 2024 and is expected to surpass 410,000 t in 2025; the regional brand “Jiashi Xinmei” is also rapidly gaining recognition [3,4]. However, along with rapid planted area expansion, fruit diseases have become increasingly prominent and now constrain the sustainable development of this industry.

Kashi prefecture has a typical arid continental climate, with hot summers, low annual precipitation, and relative humidity generally between 40% and 50%. Local plum orchards rely mainly on drip or micro-sprinkler irrigation to sustain fruit production under these dry conditions. In this region, brown spot symptoms typically begin to appear from mid-June during the rapid fruit-enlargement stage (when fruit begins to swell rapidly and change color). These production and climatic characteristics provide a context for disease development and underline the need for systematic pathogen investigation in Xinjiang orchards.

During the pre-harvest stage, various fungi can infect plum fruit and cause diseases. Among them, brown rot (caused by species such as *Monilinia fructigena*, *M. laxa*, and *M. fructicola*) is one of the most serious, and often leads to fruit rot, mummified fruit, and severe yield losses [5,6,7]. Recent molecular-level studies have offered insights into interactions between plum and brown-rot pathogens. For instance, using transcriptomics, Antanyniene et al. [8] demonstrated that plum significantly activates defense-related genes within 24–72 h after infection by *M. fructigena*. In a subsequent genome-wide association study on *P. domestica* [9], they also identified single-nucleotide polymorphisms linked to brown-rot resistance, thereby providing molecular tools for resistance breeding. In addition to these brown-rot pathogens, anthracnose caused by *Colletotrichum* spp. is also becoming an increasing problem in several *Prunus*-growing regions. Studies in China have reported different *Colletotrichum* species to coexist in various production regions and host cultivars, and for some isolates to display marked differences in fungicide sensitivity [10,11]. Post-harvest losses are also heavily driven by gray (*Botrytis cinerea*) and blue (*Penicillium expansum*) molds. Zhang et al. [12] demonstrated that salicylic acid treatment significantly enhances the antioxidant capacity of French plums and extends their shelf life. Sepulveda et al. [13] confirmed that certain antagonistic yeast strains provided effective control against gray mold.

The emergence of novel pathogens complicates the plum disease landscape. Song et al. [14] described *Mucor xinjiangensis* from Xinjiang, which causes a brown-rot-like fruit rot during the growing season; field symptoms begin as tiny red spots that enlarge into brown necrotic lesions and finally induce premature fruit drop. Aside from traditional brown-rot pathogens in the genus Monilinia, several Alternaria species (e.g., *A. alternata* and *A. arborescens* species complexes) have been reported in association with stone-fruit diseases in multiple production regions worldwide, including Europe and Asia [15,16,17]. These fungi can induce necrotic or rot symptoms during fruit development that closely resemble brown rot, frequently leading to field misidentification [18,19]. These *Alternaria* spp. also produce mycotoxins such as tenuazonic acid and alternariol that pose a potential threat to fruit quality and food safety [20]. However, the systematics of *Alternaria* remain highly contentious: more than 360 species have been described and assigned to 29 sections, and taxonomic revisions have been continuous [21]. Among them, small-spored *Alternaria* (sect. *Alternaria*) and the traditional *A. alternata* species complex (AASC) share almost identical morphologies, so their boundaries are blurred and accurate identification based solely on morphology is fraught with difficulty. Woudenberg et al. [18] reconstructed the phylogeny of this section using multilocus sequences, consolidating it into 11 phylogenetic species and one species complex, and recommended using multiple gene markers for molecular identification. Multilocus phylogenetic surveys continue to uncover cryptic diversity within *Alternaria*. For example, Nwe et al. [22] used a five-gene dataset to identify and formally describe several novel *Alternaria* species in China. These studies demonstrate that multilocus phylogenetics can clarify the systematic position and species boundaries within *Alternaria*.

In addition to fungal diseases, bacteria and viruses also threaten plum production. Bacterial spot caused by *Xanthomonas arboricola* pv. *pruni* (Xap) and plum pox virus (PPV, ‘Sharka’) can both severely compromise fruit quality and yield [23,24]. However, compared with bacterial and viral diseases, systematic research on fungal fruit diseases of plum lags; in particular, the taxonomy and phylogenetic relationships of the emerging fungal genus *Alternaria* are insufficiently studied.

The rapid expansion of the plum industry is challenged by multi-pathogen co-infection and new disease emergence. Because limited systematic research has been performed on emerging pathogens such as *Alternaria* on plum, we identify the causal agents associated with brown spot disease in Kashi, Xinjiang, determine their phylogenetic relationships using multilocus sequence analyses, and assess their pathogenicity on plum. The resulting data lay a foundation for subsequent taxonomic resolution, epidemiological investigations, and the development of effective disease-management strategies for regional plum production.

## 2. Materials and Methods

### 2.1. Sample Collection and Pathogen Isolation

**Figure 1:**
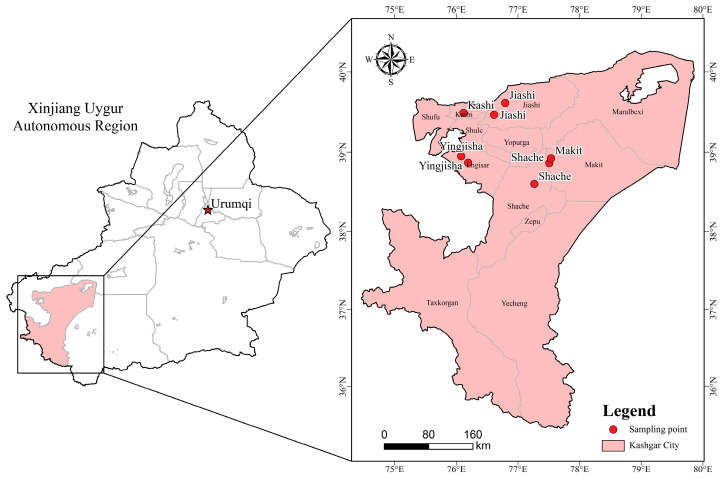
Distribution of sampling sites for brown spot-diseased plum in Kashi prefecture, Xinjiang. Red dots indicate orchard locations where symptomatic fruit were collected.

From 2023 to 2025, continuous annual surveys were performed in plum orchards in Jiashi, Shache, Yingjisha, Kashi city, and Maigaiti counties of Kashi prefecture, Xinjiang (Figure 1). In each county, each year, 1 or 2 orchards (≥5 ha) were randomly selected for sampling during the late-June phase of rapid fruit enlargement and color change. Within each orchard, 10 representative trees were chosen by five-point sampling; five symptomatic fruits showing typical brown spot lesions were collected per tree. Fruits were placed in clean kraft paper bags, labelled with collection date and GPS coordinates, transported to the laboratory, and processed within 24 h.

Pathogen isolation followed standard tissue-separation procedures: fruit surfaces were first rinsed under running water to remove dust, surface-sterilized by immersing in 70% (*v*/*v*) ethanol for 30 s and then in 1% (*w*/*v*) NaOCl for 30–60 s, rinsed three times with sterile distilled water, and blotted dry on sterile filter paper [25,26]. Inside a laminar-flow hood, 3–5 small pieces (2–5 mm) were excised from the symptomatic border with a sterile scalpel and placed onto potato dextrose agar (PDA; Beijing Solarbio Science & Technology Co., Ltd., Beijing, China). Plates were incubated at 28 °C in darkness for 5–7 days and examined daily for colony development [27]. Fungal colonies emerging from infected tissue were purified by hyphal-tip isolation; the procedure was repeated twice to obtain pure cultures. Each purified isolate was then transferred to PDA slants, incubated at 28 °C for 5 days, and then stored at 4 °C [28]. Isolation results were used to calculate the isolation frequency of each fungal taxon and for dominant species identification.

### 2.2. Morphological Observation of Isolates

Representative *Alternaria* isolates (selected from different samples showing similar colony and conidial morphology) were inoculated onto PDA plates and incubated at 28 °C in darkness for 5–10 days. Colony photographs were taken on incubation days 5 and 10, and growth rate (mm d^−1^) and morphology (colony color, diameter, margin shape, and texture) were recorded simultaneously [29]. All 210 isolates obtained in this study were initially screened for colony characteristics, and 33 isolates representing the observed morphological types were further examined microscopically. For observation of sporulation structures and conidial morphology, a light–dark alternation induction method was used: after inoculation on PDA, the isolate was first incubated at 28 °C in darkness for 4 days, then transferred to a 12/12 h light/dark cycle to promote sporulation [30]. After 7–10 days of incubation, sporulation and conidial morphology were examined microscopically. Hyphae bearing conidia at the colony margin were gently lifted and mounted on glass slides in a drop of sterile water for temporary mount preparation; observations were made using an Olympus BX43 light microscope (Olympus, Tokyo, Japan). For each representative isolate, a sample of 30–50 mature conidia was chosen at random, and their length and width were measured; diagnostic characters (shape, color, number of transverse and longitudinal/oblique septa, presence and morphology of the apical beak, and other critical traits) were recorded [31]. Preliminary species identification of isolates was performed following morphological criteria established for *Alternaria* by Simmons [32].

### 2.3. DNA Extraction and Multilocus Sequence Amplification

Representative *Alternaria* strains were grown on PDA at 28 °C in darkness for 3 d; mycelia were then harvested by gently scraping the actively growing mycelium from the colony surface. Genomic DNA was extracted using the Fungal Genomic DNA Rapid Extraction Kit (Sangon Biotech, Shanghai, China) following manufacturer’s instructions [33]. Five gene/locus regions were amplified using previously reported primer pairs: the nuclear small subunit (SSU) rRNA, large subunit (LSU) rRNA, internal transcribed spacer (ITS), Alternaria major allergen (Alt a1), and endo-polygalacturonase(endoPG) [34,35,36,37]. Each 25.0 μL PCR mixture contained 12.5 μL 2× Taq Plus MasterMix II (Sangon Biotech (Shanghai) Co., Ltd., Shanghai, China), 1.0 μL of each forward and reverse primer, 2.0 μL DNA template, and 8.5 μL ddH_2_O [38]. The PCR amplification protocol consisted of an initial denaturation at 94 °C for 4 min, followed by 35 cycles of denaturation at 94 °C for 30 s, annealing at 55 °C (ITS, endoPG), 62 °C (Alt a 1) or 59 °C (SSU, LSU) for 30 s, and extension at 72 °C for 30 s (ITS, endoPG), 45 s (Alt a 1) or 60 s (SSU, LSU), with a final extension at 72 °C for 10 min. Amplicons were examined on 1.0% agarose gels and purified using the TIANquick Midi Purification Kit (Tiangen Biotech, Beijing, China), then submitted to Tsingke Biological Technology Co., Ltd. (Xi’an, China) for bidirectional Sanger sequencing. Resulting sequences were manually edited and trimmed using BioEdit version 7.0.0, queried against the NCBI nucleotide (nt) database using the BLAST+ BLASTn algorithm (https://ftp.ncbi.nlm.nih.gov/blast/executables/blast+/LATEST/?referrer=grok.com, 3 September 2025), and then submitted to GenBank.

### 2.4. Phylogenetic Analysis

To clarify phylogenetic relationships among isolated strains, both single-gene (ITS) and multigene (ITS, SSU, LSU Alt a1 and endoPG) analyses were performed. First, an ITS-based phylogeny was constructed. After alignment with MAFFT v.7.450 and manual adjustment, a Maximum-Likelihood (ML) tree was generated in MEGA v.7.0 [39]. Phylogenetic analysis was performed using the Tamura 3-parameter (T92) + Gamma (+G) model, which was selected as the best-fit nucleotide substitution model based on model testing in MEGA, with 1000 bootstrap replicates to assess branch support. The resulting tree provided an initial estimate of each isolate’s phylogenetic placement and its relatedness to reference strains.

To further resolve phylogenetic positions of representative *Alternaria* isolates, the five-locus dataset (ITS, SSU, LSU, Alt a1 and endoPG) was concatenated and analyzed jointly. These sequences were aligned with corresponding sequences of relevant *Alternaria* ex-type strains downloaded from the NCBI GenBank (https://www.ncbi.nlm.nih.gov/genbank/, accessed on 13 October 2025) [18]. GenBank accession numbers of all isolates and reference strains used in phylogenetic analyses are summarized in Table 1 (see also Supplementary Table S1 for detailed ITS sequence information). Each gene region was aligned individually with MAFFT v.7.427, manually edited in MEGA v.7.0, and then concatenated into a single multilocus dataset using SequenceMatrix v.1.8 [40]. The optimal substitution model was then selected with ModelFinder under the Bayesian Information Criterion [41]. Multilocus phylogenetic analysis was performed with MrBayes v.3.2.7 under Bayesian inference. The GTR + I + G model was specified; four independent Markov chains were run for 1,000,000 generations, sampling every 100 generations, and the first 10% of samples were discarded as burn-in for posterior-probability (PP) calculation. The resulting tree was visualized and annotated in FigTree v.1.4.4 [42]. Results were used to confirm the phylogenetic position and taxonomic placement of isolated strains.

### 2.5. Pathogenicity Test

Ten fungal isolates (KSPM 50, 135, 201, 25, 121, 12, 8, 202, 40, and 134) were selected for pathogenicity assays. Each isolate was cultured on potato dextrose agar (PDA) at 28 °C under a 12/12 h light/dark photoperiod for 10 days. To determine the pathogenicity of the isolates, ten representative strains (KSPM 50, 135, 201, 25, 121, 12, 8, 202, 40, and 134) were selected for inoculation tests. Each isolate was grown on PDA at 28 °C under a 12/12 h light/dark photoperiod for 10 d. Following Prencipe et al. [53], conidia were harvested by flooding each plate with 5 mL sterile water containing 10 µL Tween 20, gently scraping the surface to dislodge conidia, and filtering the resulting suspension through four layers of sterile cheesecloth. The collected suspension was counted with a hemocytometer and adjusted to 1 ×10^5^ conidia mL^−1^ [54]. Following Ahmad et al. [15], uniform, mechanically undamaged, and pest-free plum fruits (cv. ‘Français’) were selected for the pathogenicity assay. Fruit surfaces were disinfected by immersion in 1% (*w*/*v*) NaOCl for 1 min, rinsed three times with sterile distilled water, and air-dried for 5 min. Each isolate was tested in three biological replicates, with three fruits per replicate. Each fruit was punctured once with a sterile needle to create a wound ~1 mm in diameter, into which 1 µL of conidial suspension was deposited. Control fruits received 1 µL of sterile distilled water in the same manner to ensure identical inoculation volumes [16]. Inoculated fruits were placed in sterile plastic trays, covered with plastic film to maintain high humidity, and incubated at 28 °C. Disease development was monitored and lesion areas were measured 3 and 5 days post-inoculation (dpi). Lesion areas (mm^2^) were quantified with ImageJ v.1.53e (NIH, USA): after scale calibration (Set Scale), the “Freehand Selection” tool (https://docs.krita.org/en/reference_manual/tools/freehand_select.html, 27 October 2025) was used to outline each lesion and calculate its area. The pathogen was subsequently re-isolated from the lesion margins of diseased fruits and identified on the basis of morphology and ITS sequencing to fulfill Koch’s postulates.

### 2.6. Statistical Analysis

All statistical analyses were performed with IBM SPSS Statistics v.28.0.1.0 (IBM Corp., Armonk, NY, USA). Rot diameters obtained from the pathogenicity assay were subjected to one-way analysis of variance, with means compared using Tukey’s test at a significance threshold of *p* ≤ 0.05.

## 3. Results

### 3.1. Field Symptoms

**Figure 2:**
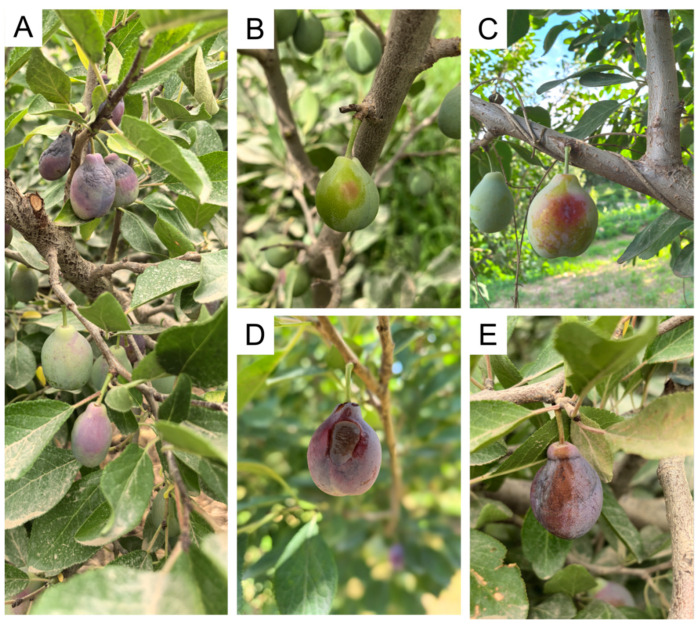
Field symptoms of brown spot disease on plum in Kashi, Xinjiang. (**A**) General disease situation in the orchard; (**B**) early stage: pin-head-sized, pale-red to light-brown spots on the fruit skin; (**C**) spots enlarge into circular or elliptical brown necrotic lesions with indistinct margins that gradually turn dark brown; (**D**) multiple lesions coalesce into large necrotic areas, accompanied by slight depression and shrinkage of the fruit surface; and (**E**) late stage: necrotic tissue markedly sinks, dries and hardens.

Orchards often bore fruit at different disease stages simultaneously (Figure 2A). The earliest symptoms were pin-head-sized, pale-reddish to light-brown spots of 0.5–1.5 mm diameter, scattered on the peel, especially on the flanks or sun-exposed sides; the skin had lost its natural gloss (Figure 2B). As the disease progressed, spots enlarged into circular or elliptical brown necrotic lesions 5–8 mm across with indistinct margins that graded from light to dark brown; some turned brown-black (Figure 2C). Further development resulted in individual lesions coalescing into large, irregular necrotic areas accompanied by slight peel depression and shrinkage; the necrotic tissue remained firm (Figure 2D). During the late stage, necrotic zones markedly sunk, dried, and hardened; the entire fruit lost water, turned dark-brown to almost black, and either dropped prematurely or remained on the tree in a mummified condition (Figure 2E).

### 3.2. Cultural Characteristics and Systematic Classification of Brown Rot Isolates

**Figure 3:**
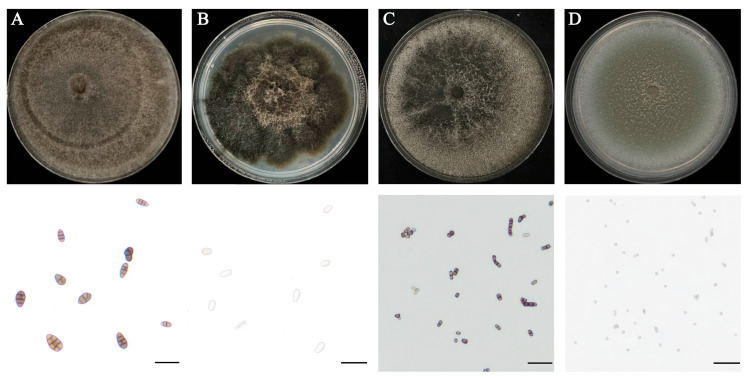
Colony (upper) and conidial (lower) morphology of different fungal isolates on PDA medium. (**A**) *Alternaria* sp. (KSPM 50); (**B**) *Diplodia* sp. (KSPM 296); (**C**) *Neoscytalidium* sp. (KSPM 6); (**D**) *Aspergillus* sp. (KSPM 104). Colonies were grown on PDA for 10 days at 28 °C in the dark, and conidia were observed under light microscopy (Scale bars = 20 μm).

A total of 210 fungal strains were isolated from spot lesions on fruit. Macroscopic examination revealed marked inter-strain differences in colony texture, color, and conidial morphology (Figure 3). Based on morphology, the predominant fungi were assigned to one of *Alternaria*, *Diplodia*, *Neoscytalidium*, or *Aspergillus*.

*Alternaria* isolates produced villous or felt-like colonies that were dense and initially white to light olive-green, later turning dark olive-green to brownish-green; faint concentric zones were visible on the surface of some cultures. Microscopically, conidia were pale to mid-brown, ovoid or obclavate, 1–7-transversely septate and 0–3-longitudinally septate, and had a short beak at the apex of some spores (Figure 3A). Colonies of *Diplodia* were dense, with a rough surface and frequently raised center. Their color gradually changed from gray to dark gray or blackish brown. Conidia were ovoid to elliptical, with thick walls; they were initially hyaline but became dark brown upon maturation (Figure 3B). Isolates of *Neoscytalidium* grew rapidly; colonies shifted from light grey to dark grey or nearly black and often developed radial fissures or fan-shaped sectors. Microscopically, arthroconidia were short-cylindrical to rectangular and usually arranged in chains (Figure 3C). *Aspergillus* isolates also grew fast and produced powdery or finely velvety colonies that were grey-green; numerous globose conidia were observed microscopically (Figure 3D).

**Figure 4:**
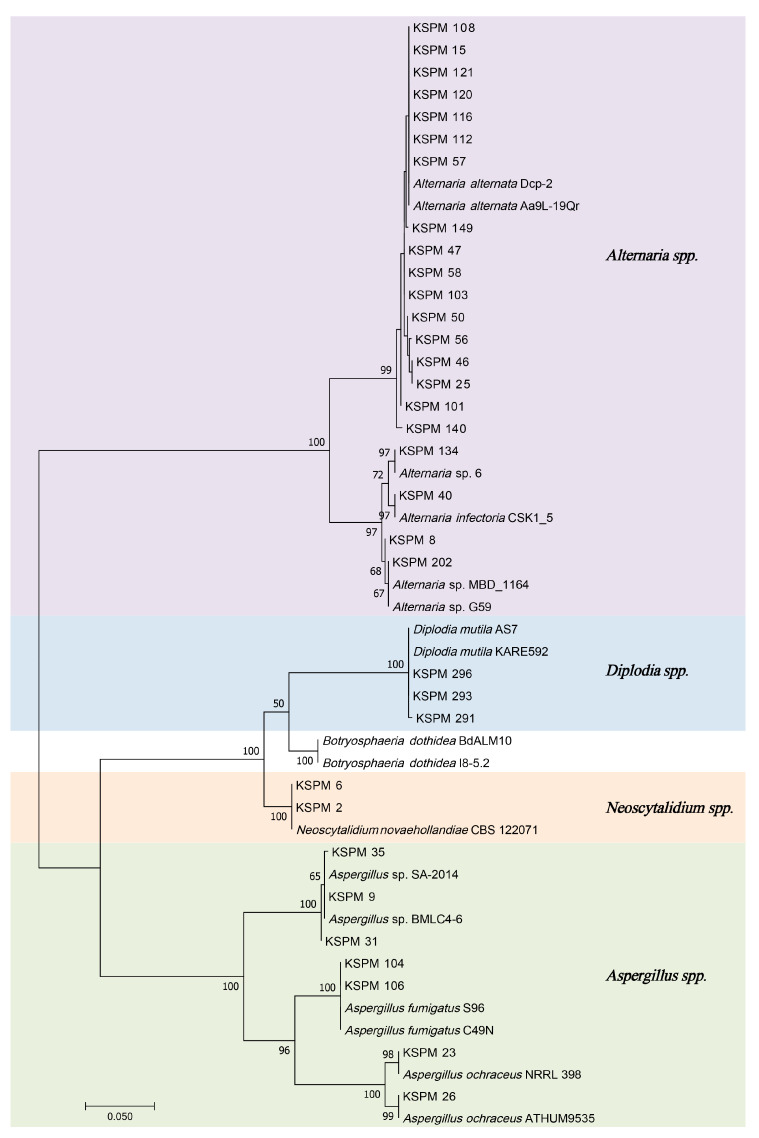
Maximum likelihood phylogenetic tree based on ITS sequences of fungal isolates obtained from symptomatic plum fruits. Bootstrap values (>50%) from 1000 replications are shown at branch nodes. The tree was rooted using midpoint rooting. Strains isolated in this study are labeled with isolate codes (KSPM series). Major clades corresponding to *Alternaria*, *Diplodia*, *Neoscytalidium*, and *Aspergillus* are shaded in different colors. The scale bar indicates 0.05 substitutions per nucleotide position.

To corroborate morphological identifications and clarify systematic relationships, ITS sequences of 33 representative isolates were subjected to ML phylogenetic analysis. The resulting tree (Figure 4) resolved all isolates into four well-supported clades that correspond to the genera *Alternaria*, *Diplodia*, *Neoscytalidium*, and *Aspergillus*. Each clade clustered with its respective ex-type or reference strain, with bootstrap support for all inter-generic nodes (mostly ≥80%), indicating robust phylogenetic delineation among genera.

Based on ITS-based clustering and morphology, *Alternaria* was the dominant taxon, accounting for 84.8% (178/210) of all isolates; *Aspergillus* (20), *Diplodia* (7), and *Neoscytalidium* (5) followed.

**Figure 5:**
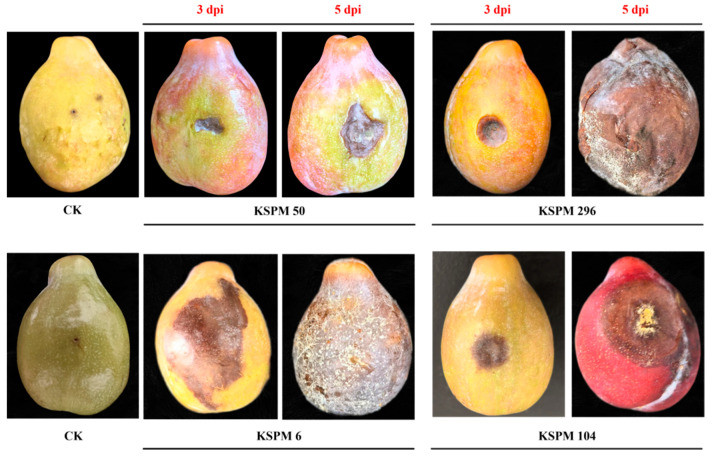
Pathogenicity of fungal isolates on plum fruit. Healthy fruits were wounded and inoculated with representative strains (KSPM 50 (*Alternaria* spp.), KSPM 296 (*Diplodia* spp.), KSPM 6 (*Neoscytalidium* spp.), and KSPM 104 (*Aspergillus* spp.), then incubated in a moist chamber at 28 °C CK represents the uninoculated control. Photographs were taken at 3 and 5 days post-inoculation to document lesion development.

Pathogenicity tests on detached plum fruits under wounded conditions revealed all four genera to be capable of inducing lesion development, although symptom characteristics varied. *Alternaria* strains produced circular to irregular, light- to dark-brown spots with sharply defined margins that became slightly sunken at advanced stages—symptoms identical to typical brown spot lesions observed in the field. In contrast, *Aspergillus*, *Diplodia*, and *Neoscytalidium* isolates caused rapid, soft-rot or general mushy decay; lesions expanded quickly, lacked clear boundaries, and led to extensive tissue liquefaction symptoms, clearly deviating from those in the field (Figure 5).

Integrating morphological observations and molecular phylogenetic analyses, we conclude that *Alternaria* is the most abundant taxon within the fungal community. Pathogenicity was demonstrated under artificial inoculation conditions by representative isolates. Consequently, *Alternaria* isolates were selected for subsequent multilocus phylogenetic analyses, with pathogenicity assays performed on representative strains to clarify species composition and systematic position.

### 3.3. Phylogenetic Analysis

**Figure 6:**
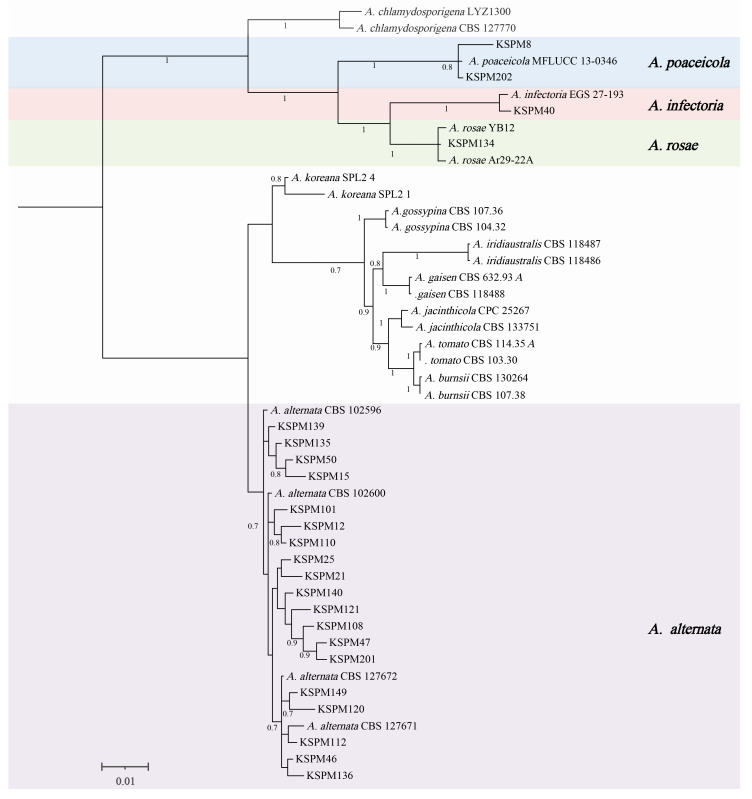
Phylogenetic tree of *Alternaria* isolates. Bayesian inference tree constructed from the concatenated sequences of ITS, SSU, LSU, Alt a1 and endoPG. Numbers at nodes are Bayesian posterior probabilities; only values ≥0.70 are shown. The scale bar indicates the number of nucleotide substitutions per site.

Based on morphology and ITS barcoding, 23 representative isolates were selected for multilocus phylogenetic analysis. A Bayesian inference tree constructed from the concatenated five-gene dataset (ITS, SSU, LSU, Alt a1, and endoPG) is shown in Figure 6. All KSPM isolates obtained from symptomatic Xinjiang plums fell within the small-spored *Alternaria* clade, forming several well-supported sub-clades (PP = 0.7–1.0). Most isolates (KSPM 25, 21, 110, 50, 108, 47, 12, 140, 139, 101, 46, 136, 149, 135, 121, 120, 15, 112, and 201) grouped into a single, highly supported lineage together with the ex-type and reference strains of *A. alternata* (CBS 102600, 127671, 127672, 102596), constituting the *A. alternata* species complex (AASC) sensu Woudenberg et al. (2015) [18]. Although minor sequence variation was detected among isolates, the clade remained topologically stable and strongly supported, confirming their placement within the AASC.

A few isolates occupied positions outside the core *A. alternata* clade: KSPM 202 and KSPM 8 clustered with *A. poaceicola* (MFLUCC 13-0346; PP = 0.8), KSPM 134 allied with the *A. rosae* lineage (strains YB12, Ar29-22A; PP = 0.7–1.0), and KSPM 40 formed a fully supported branch (PP = 1.0) with *A. infectoria* (EGS 27-193). These data indicate that, in addition to the dominant AASC, plum brown spot lesions in Xinjiang also harbor *Alternaria* taxa related to *A. poaceicola*, *A. rosae*, and *A. infectoria*. Overall, all major nodes had a high PP (≥0.7), yielding a robust topology.

### 3.4. Morphological Characterization

**Figure 7:**
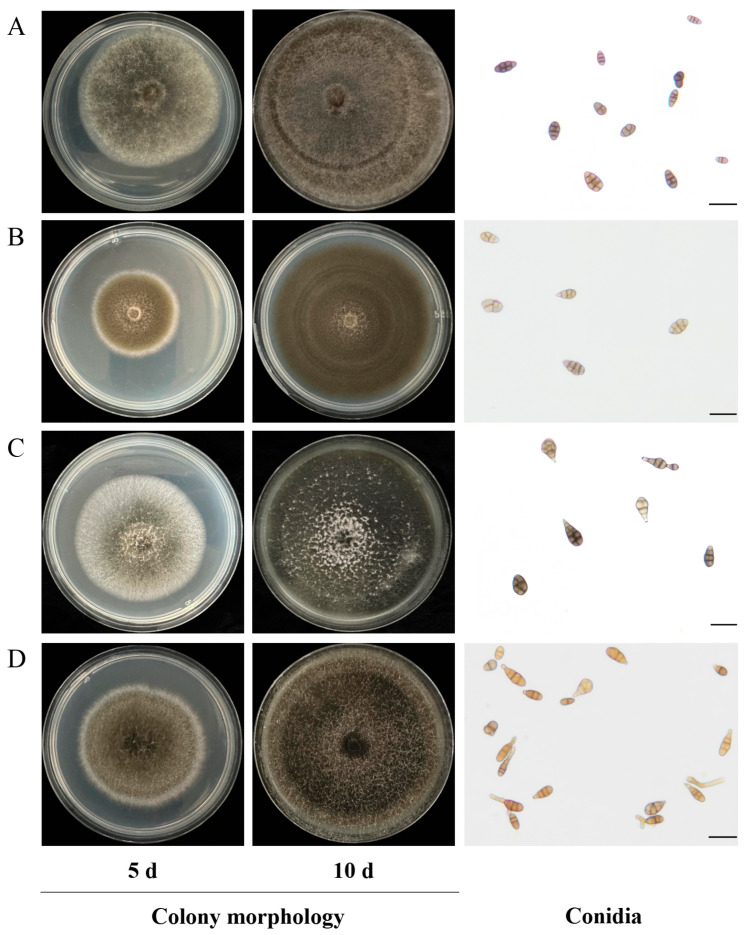
Cultural and microscopic morphology of representative *Alternaria* isolates. A–D correspond to: (**A**) *A. alternata* KSPM 50, (**B**) *A. infectoria* KSPM 40, (**C**) *A. poaceicola* KSPM 8, and (**D**) *A. rosae* KSPM 134. For each row, images from left to right show colony morphology after 5 and 10 days of incubation, followed by the corresponding micrograph of conidia (scale bar = 20 µm).

Four representative *Alternaria* isolates (KSPM 50, 40, 8, 134) were plated on PDA and incubated at 28 °C under a 12 h light/dark photoperiod. After 7–10 days, all strains exhibited a typical phenotype of small-spored *Alternaria* (sect. *Alternaria*) [18] (Figure 7).

*A. alternata* KSPM 50 (sect. *Alternaria*, AASC): Colonies on PDA grew rapidly, initially white to pale olive-green and cottony; after 7–10 days the center turned dark green to dark brown, often with faint concentric rings. Reverse colony pigmentation was dark brown, with no diffusible pigment. Conidiophores were short to medium in length and produced mostly short acropetal chains. Conidia were obclavate to long-ellipsoid, pale to mid-brown, smooth or slightly rough-walled, aseptate to 7-transversely septate and 0–4-longitudinally septate, and mostly beakless or with a short beak. Spore size was (6.9–)12.4–17.7(–20.8) × (4.2–)5.0–7.8(–8.5) µm (Figure 7A). These traits match the typical description of small-spored *A. alternata* [55].

*Alternaria infectoria* KSPM 40 (sect. *Infectoriae*): colonies were cottony, white at first, turning pale-green to dark green in the center with age; concentric rings were indistinct. Reverse colony pigmentation was pale to dark brown, with no diffusible pigment. Conidiophores were mostly short-chained or solitary, with occasional secondary short chains formed at hyphal tips. Conidia were ovoid to short-ellipsoid, light brown, smooth-walled, beakless or with a very short beak, with 1–3 transverse and 0–3 longitudinal septa; spore size was (6.1–)13.5–20.0(–36.7) × (4.1–)7.4–11.9(–14.3) µm (Figure 7B). These traits correspond to the *A. infectoria* group, characterized by relatively short, wide, pale conidia with few septa and occasional secondary chains [56].

*Alternaria poaceicola* KSPM 8 (sect. *Alternaria*): colonies were erect-villous, white at first; the center later turned yellow-green to dark green, with scant sporulation. Reverse colony pigmentation was pale brown, with no diffusible pigment. Conidiophores were short, borne singly or in short chains. Conidia were cylindrical to long-ellipsoid, pale brown, smooth-walled, mostly beakless or with a short beak, with 1–5 transverse and 0–3 longitudinal septa; spore size was (5.3–)7.0–18.8(–22.7) × (4.1–)5.0–8.9(–9.6) µm (Figure 7C). This morphology matches the ex-type strain CBS 140208 of *A. poaceicola* [46].

*Alternaria rosae* KSPM 134 (sect. *Pseudoalternaria*): colonies were felty-reticulate, white to pale grey at first, with the center later shifting from grey-green to dark green, with low sporulation. Reverse colony pigmentation was brown, with no diffusible pigment. Conidiophores were short, arising singly or in short chains. Conidia were cylindrical to narrowly ellipsoid, pale to mid-brown, slightly roughened, mostly beakless or with a short beak, with 1–5 transverse and 0–3 longitudinal septa; spore size was (7.1–)8.0–16.0(–21.2) × (3.3–)4.3–9.0(–12.0) µm (Figure 7D). These morphological traits agree with the original description of the ex-type strain CBS 102.93 of *A. rosae* [57].

### 3.5. Pathogenicity Verification

**Figure 8:**
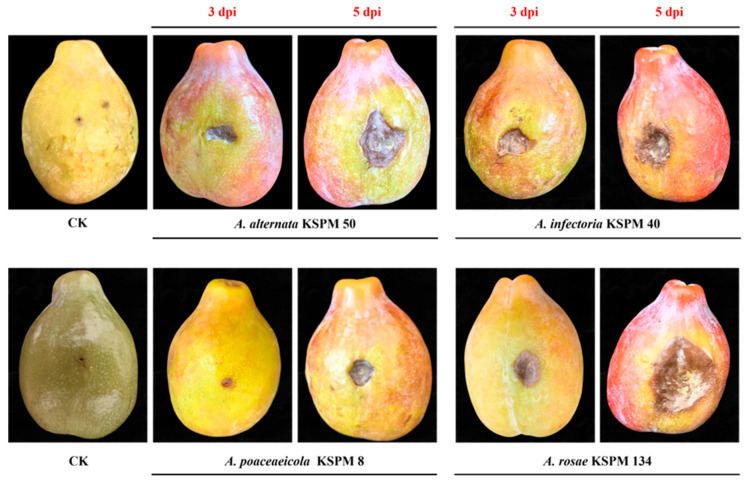
Pathogenicity of *Alternaria* isolates on plum fruit. CK = non-inoculated control (photographed at 5 dpi). Fruit inoculated with *A. alternata* KSPM 50, *A. infectoria* KSPM 40, *A. poaceicola* KSPM 8 and *A. rosae* KSPM 134 developed small necrotic spots at 3 dpi that expanded into brown necrotic lesions by 5 dpi.

Pathogenicity assays were conducted using ten representative *Alternaria* isolates. Necrotic lesions were observed on inoculated plum fruits at 3 dpi, and lesions gradually expanded with disease progression. At the early stage, small, light gray-brown necrotic spots appeared at the inoculation sites, while control fruits inoculated with sterile distilled water remained symptomless throughout the experiment (Figure 8). With increasing incubation time, lesion expansion was evident on all inoculated fruits. By 5 dpi, lesions had developed into brown to dark-brown necrotic areas, often sunken and irregular in shape. All ten isolates induced visible necrotic lesions by 5 dpi, and symptom development was consistent with brown spot symptoms observed under field conditions (Figure 8).

**Figure 9:**
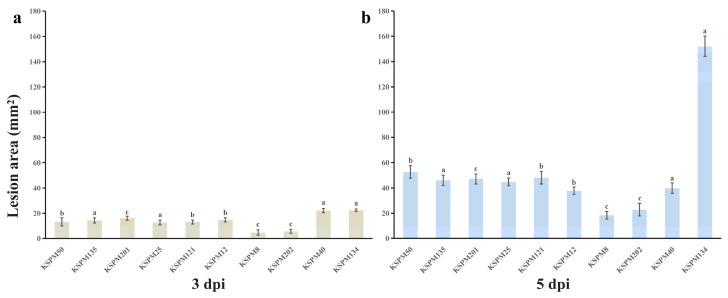
Differential virulence of *Alternaria* isolates on plum fruit. (**a**) Mean lesion area (mm^2^) on healthy plum fruit at 3 dpi and (**b**) at 5 dpi. CK (non-inoculated control) showed no lesion development at either 3 or 5 dpi. Different letters above bars indicate significant differences among isolates at the same time point (Tukey’s HSD, *p* < 0.05).

Quantitative analysis revealed significant differences in lesion area among isolates (*p* < 0.05) (Figure 9). At 3 dpi, mean lesion areas produced by KSPM 40 (22.2 mm^2^) and KSPM 134 (22.4 mm^2^) were significantly larger than those caused by KSPM 50 (13.2 mm^2^) and KSPM 8 (4.9 mm^2^) (Figure 9a). At 5 dpi, lesion areas increased for all isolates, with KSPM 134 producing the largest lesions (152.0 mm^2^), followed by KSPM 50 (52.6 mm^2^), KSPM 40 (39.8 mm^2^), and KSPM 8 (18.3 mm^2^) (Figure 9b). Fungal isolates were consistently recovered from the margins of necrotic lesions on inoculated fruits, and the recovered isolates were identical to the original inoculated strains based on colony morphology and ITS sequence analysis.

## 4. Discussion

Through continuous monitoring of plum brown spot samples collected in the main production areas of Kashi, Xinjiang, from 2023 to 2025, and by concatenated phylogenetic analyses of five loci, we confirm that AASC is the dominant causal agent of this disease. Regardless of year or orchard, *Alternaria* was recovered from >80% of symptomatic fruits—a frequency significantly higher than that of any other fungus. Consistently, multigene phylogenetic analyses revealed minimal sequence divergence between the majority of isolates and the *A. alternata* ex-type strain, further indicating that *A. alternata* was the primary causal agent of brown spot on plum in Xinjiang. Not only is this the first definitive evidence that *Alternaria* species are the primary pathogens responsible for plum brown spot in the major production regions of Kashi, but it is also an important addition to the pathogen spectrum of *Prunus* crops in China.

We did not detect the fungus *M. xinjiangensis*, which was reported by Song et al. [14], to cause plum fruit brown rot. This indicates an ecological niche differentiation in the pathogen complex associated with plum fruit diseases in the Kashi region. Whereas *Alternaria* spp. typically induce dry, necrotic lesions confined to the peel, mucor-like pathogens predominantly cause wet, spongy rot that rapidly invades the fruit pulp [58,59]. This contrast suggests that plum brown spot and brown rot are driven by distinct ecological guilds, as reflected by the clearly different symptom types and pathogen isolation patterns that we report; mechanisms of competition or interaction among these pathogens warrant further investigation. Collectively, the observed consistency and divergence are consistent with previous reports that link dominant fruit pathogens with climatic moisture regimes. This implies that within the arid oasis to semi-arid orchard ecosystem of Kashi, Xinjiang, the assembly of dominant fruit pathogens is shaped by a combination of regional climate, cultivation practices (irrigation, fertilization, pruning), cultivar composition, and fungicide-use history, giving rise to a location-specific pathogen community structure.

*Alternaria* species are ubiquitous in nature, and many are important plant pathogens. *A. alternata*, a cosmopolitan species complex, infects a wide range of fruit trees and field crops. Waqas et al. [16] used a four-gene concatenated dataset to identify the *A. alternata* complex as the predominant causal agent of sweet cherry black rot across multiple years and orchards in Italy, underscoring its stable pathogenicity and strong adaptive capacity across regions. Investigations of apple leaf spot and fruit spot have frequently treated several *Alternaria* taxa (e.g., *A. alternata*, *A. arborescens*, and *A. tenuissima*) as members of a single species complex, indicating that the small-spored *Alternaria* group has a broad virulence spectrum and intricate host associations within tree fruit production systems [60]. At the same time, *A. alternata* can cause brown rot of citrus and black spot of persimmon and other fruit diseases [61,62]. In Europe, *Alternaria* is also involved in rot symptoms of stone fruits such as plums [16]. However, in China, *Alternaria* has only recently been reported to infect plum fruits [16]. Our study demonstrates that *Alternaria* acts as the primary pathogen causing plum brown spot in Xinjiang, and we clarify its taxonomic status.

Based on multigene sequence analysis, we classify the main pathogen of plum brown spot disease in the AASC. This complex includes many small-spored *Alternaria* species with highly similar morphological and genetic characteristics that have traditionally been collectively referred to as “broad-sense *A. alternata*.” Recent multi-locus phylogenetic studies have revealed multiple independent evolutionary lineages within the AASC. Li et al. [21] divided the genus *Alternaria* into several sections (sect.) and pointed out that the AASC may contain several cryptic species or evolutionary lineages. He et al. [63] identified seven new species in this complex on leaves of Chinese fir, further demonstrating its complex genetic differentiation. Our strains are morphologically indistinguishable from type strains, but they form independent branches in the multigene phylogenetic tree, reflecting the extensive genetic variability that has been widely reported within the *A. alternata* species complex [18,21,55].

In addition to the AASC, we sporadically isolated *Aspergillus* spp., *Diplodia mutila*, and *Neoscytalidium dimidiatum*. Although these fungi can induce fruit rot under artificial inoculation, their infection sites, lesion characteristics, and ecological preferences differ markedly from the dry, superficial brown spots typical of AASC-associated disease.

*Aspergillus* spp. are common post-harvest pathogens that cause water-soaked, rapidly expanding soft rot [64]. These symptoms contrast sharply with the dry, sharply margined lesions caused by *Alternaria*. *Diplodia* species are opportunistic wound pathogens with a wide host range [65,66]. Our *Diplodia* isolate induced localized dry rot when inoculated through wounds, a pattern inconsistent with the naturally occurring AASC lesions observed on intact plum fruit. *Neoscytalidium dimidiatum* is a thermotolerant, drought-adapted opportunist reported in many woody crops worldwide [67,68]. Although a few plum fruits showed dry-rot symptoms resembling those caused by this species, its sporadic isolation and wound-associated infection suggest that it plays only a minor role under field conditions. These secondary fungi differ markedly from *Alternaria* in their symptoms, infection pathways, and ecological preferences, and their isolation frequency was very low. This clear divergence further indicates that the AASC is the primary causal agent of plum brown spot in the Kashi region, and other fungi function only as occasional opportunistic pathogens, becoming relevant mainly under post-harvest or extreme environmental conditions.

In finding *Alternaria* to be the main cause of plum brown spot, established control tactics developed against it and related small-spored species can be used in its treatment. In the orchard, mummified and fallen fruit should be promptly removed and destroyed, trees should be pruned to improve air circulation and light penetration, and canopy humidity should be kept low to reduce primary inocula. For chemical control, Song et al. [14] reported difenoconazole to provide the best efficacy against mucor-induced brown rot of plum, and recommended it as one of the first-choice fungicides. Combinations of strobilurins (e.g., azoxystrobin), imidazoles (e.g., prochloraz), and dithiocarbamates (e.g., mancozeb) have shown synergistic activity against *Alternaria* diseases in many crops; rotating or tank-mixing these modes of action can slow resistance development [69]. Post-harvest, fruit should be kept dry and stored at low temperature to suppress latent infections. Integrating cultural and chemical tactics will help build a sustainable management system for plum diseases.

## 5. Conclusions

We clarify the principal pathogen of plum brown spot in Kashi and its ecological diversity. This provides a scientific basis for establishing regional disease monitoring and green-management systems. Future work could investigate the pathogen’s infection mechanisms, mycotoxin production, and fungicide resistance evolution, while screening highly effective, low-toxicity compounds and resistant cultivars to support the sustainable development of Xinjiang’s prune industry.

## Figures and Tables

**Table 1:** Detailed information and GenBank accession numbers of Alternaria species used in the phylogenetic analysis.

Species	Strain	Host	Country	GenBank Accession Number					
				ITS	LSU	SSU	Alt a1	endoPG	References
*Alternaria alternata*	CBS 102600	*Citrus reticulata*	USA	KP124331	KP124483	KP124953	KP123880	KP124033	[18]
*A. alternata*	CBS 127671	*Stanleya pinnata*	USA	KP124381	KP124535	KP125005	KP123929	KP124085	[18]
*A. alternata*	CBS 127672	*Astragalus bisulcatus*	USA	KP124382	KP124536	KP124382	KP123930	KP124086	[18]
*A. alternata*	CBS 102596	*Citrus jambhiri*	USA	KP124328	KP124480	KP124950	KP123877	KP124030	[18]
*A. burnsii*	CBS 107.38	*Cuminum cyminum*	India	KP124420	KP124573	KP125043	KP123967	KP124124	[18]
*A. burnsii*	CBS 130264	*human sputum*	India	KP124425	KP124578	KP125048	KP123972	KP124129	[18]
*A. tomato*	CBS 103.30	*Solanum lycopersicum*	Unknown	KP124445	KP124599	KP125069	KP123991	KP124151	[18]
*A. tomato*	CBS 114.35	*Solanum lycopersicum*	Unknown	KP124446	KP124600	KP125070	KP123992	KP124152	[18]
*A. jacinthicola*	CBS 133751	*Eichhornia crassipes*	Mali	KP124438	KP124592	KP125062	KP123984	KP124143	[18]
*A. jacinthicola*	CPC 25267	*Cucumis melo* var. inodorus	Unknown	KP124439	KP124593	KP125063	KP123985	KP124144	[18]
*A. gaisen*	CBS 118488	*Pyrus pyrifolia*	Japan	KP124427	KP124581	KP125051	KP123975	KP124132	[18]
*A. gaisen*	CBS 632.93	*Pyrus pyrifolia*	Japan	KC584197	KC584275	KC584531	-	AY250933	[34,43]
*A. iridiaustralis*	CBs 118486	*Iris* sp.	New Zealand	KP124434	KP124588	KP125058	KP123981	KP124140	[18]
*A. iridiaustralis*	CBS 118487	*Iris* sp.	Australia	KP124436	KP124590	KP125060	KP123982	KP124141	[18]
*A. gossypina*	CBS 104.32	*Gossypium* sp.	Zimbabwe	KP124430	KP124584	KP125054	JQ646419	KP124015	[18,44]
*A. gossypina*	CBS 107.36	soil	Indonesia	KP124431	KP124585	KP125055	-	KP124136	[18]
*A. koreana*	SPL2-1	*Atractylodes ovata*	Republic of Korea	LC621613	-	-	LC631831	LC631844	[45]
*A. koreana*	SPL2-4	*Atractylodes ovata*	Republic of Korea	LC621615	LC621615	LC621615	LC631832	LC631845	[45]
*A. poaceicola*	MFLUCC 13-0346	*Dactylis glomerata*	Italy	KY026587	KY205718	KY038357	-	-	[46]
*A. malorum*	CBS 135.31	Unknown	Unknown	JQ693638	-	-	JQ646369	-	[44]
*A. malorum*	CBS 126589	*Homo sapiens*	Iran	JQ219160	MH875629		-	-	[47,48]
*A. rosae*	Ar29-22A	Meadow air	Poland	OP718455	-	-	OP758822	-	[49]
*A. rosae*	YB12	Rose petals	China	MW821473	-	-	-	-	-
*A. infectoria*	EGS 27-193	*P istachio*	USA	AF347034	AY293859	AY278846	FJ266502	-	[50,51]
*A. chlamydosporigena*	CBS 127770	Unknown	USA	MH864769	MH876209	-	-	-	[48]
*A. chlamydosporigena*	LYZ1300	Poa poophagorum	China	PV926704	PV926712	-	-	-	[52]
***A.*** ***alternata***	**KSPM 201**	***Prunus domestica***	**China**	**PX457268**	**PX457662**	**PX457685**	**PX503303**	**PX503326**	**-**
***A.*** ***alternata***	**KSPM 47**	***Prunus domestica***	**China**	**PX457282**	**PX457676**	**PX457699**	**PX503289**	**PX503312**	**-**
***A.*** ***alternata***	**KSPM 108**	***Prunus domestica***	**China**	**PX457279**	**PX457673**	**PX457696**	**PX503292**	**PX503315**	**-**
***A.*** ***alternata***	**KSPM 121**	***Prunus domestica***	**China**	**PX457275**	**PX457669**	**PX457692**	**PX503296**	**PX503319**	**-**
***A.*** ***alternata***	**KSPM 140**	***Prunus domestica***	**China**	**PX457270**	**PX457664**	**PX457687**	**PX503301**	**PX503324**	**-**
***A.*** ***alternata***	**KSPM 21**	***Prunus domestica***	**China**	**PX457286**	**PX457680**	**PX457703**	**PX503285**	**PX503308**	**-**
***A.*** ***alternata***	**KSPM 25**	***Prunus domestica***	**China**	**PX457285**	**PX457679**	**PX457702**	**PX503286**	**PX503309**	**-**
***A.*** ***alternata***	**KSPM 136**	***Prunus domestica***	**China**	**PX457272**	**PX457666**	**PX457689**	**PX503299**	**PX503322**	**-**
***A.*** ***alternata***	**KSPM 46**	***Prunus domestica***	**China**	**PX457283**	**PX457677**	**PX457700**	**PX503288**	**PX503311**	**-**
***A.*** ***alternata***	**KSPM 120**	***Prunus domestica***	**China**	**PX457276**	**PX457670**	**PX457693**	**PX503295**	**PX503318**	**-**
***A.*** ***alternata***	**KSPM 149**	***Prunus domestica***	**China**	**PX457269**	**PX457663**	**PX457686**	**PX503302**	**PX503325**	**-**
***A.*** ***alternata***	**KSPM 110**	***Prunus domestica***	**China**	**PX457278**	**PX457672**	**PX457695**	**PX503293**	**PX503316**	**-**
***A.*** ***alternata***	**KSPM 12**	***Prunus domestica***	**China**	**PX457288**	**PX457682**	**PX457705**	**PX503283**	**PX503306**	**-**
***A.*** ***alternata***	**KSPM 101**	***Prunus domestica***	**China**	**PX457280**	**PX457674**	**PX457697**	**PX503291**	**PX503314**	**-**
***A.*** ***alternata***	**KSPM 15**	***Prunus domestica***	**China**	**PX457287**	**PX457681**	**PX457704**	**PX503284**	**PX503307**	**-**
***A.*** ***alternata***	**KSPM 50**	***Prunus domestica***	**China**	**PX457281**	**PX457675**	**PX457698**	**PX503290**	**PX503313**	**-**
***A.*** ***alternata***	**KSPM 135**	***Prunus domestica***	**China**	**PX457273**	**PX457667**	**PX457690**	**PX503298**	**PX503321**	**-**
***A.*** ***alternata***	**KSPM 139**	***Prunus domestica***	**China**	**PX457271**	**PX457665**	**PX457688**	**PX503300**	**PX503323**	**-**
***A.*** ***alternata***	**KSPM 112**	***Prunus domestica***	**China**	**PX457277**	**PX457671**	**PX457694**	**PX503294**	**PX503317**	**-**
***A.*** ***poaceicola***	**KSPM 8**	***Prunus domestica***	**China**	**PX457289**	**PX457683**	**PX457706**	**PX503282**	**PX503305**	**-**
***A.*** ***poaceicola***	**KSPM 202**	***Prunus domestica***	**China**	**PX457267**	**PX457661**	**PX457684**	**PX503304**	**PX503327**	**-**
***A.*** ***rosae***	**KSPM 134**	***Prunus domestica***	**China**	**PX457274**	**PX457668**	**PX457691**	**PX503297**	**PX503320**	**-**
***A.*** ***infectoria***	**KSPM 40**	***Prunus domestica***	**China**	**PX457284**	**PX457678**	**PX457701**	**PX503287**	**PX503310**	**-**

Note: -, no sequence. New sequences are in bold.

## Data Availability

The original contributions presented in this study are included in the article/Supplementary Material. Further inquiries can be directed to the corresponding author.

## Supplementary Materials

The following supporting information can be downloaded at: https://www.mdpi.com/article/10.3390/jof12010069/s1, Table S1: Detailed information and GenBank accession numbers of isolates and reference strains used for the ITS-based single-gene phylogenetic analysis.
