# Supplementary material for: Identification and Multigene Phylogenetic Analysis Reveal Alternaria as the Primary Pathogen Causing European Plum (Prunus domestica) Brown Spot in Xinjiang, China
Source: J Fungi (Basel). 2026 Jan 15;12(1):69. doi: 10.3390/jof12010069 (PMC12843153; doi:10.3390/jof12010069)
Supplement: Supplementary file 1 [file jof-12-00069-s001.zip › jof-4018116-supplementary.pdf]

Supplementary Table S1. Detailed information and GenBank accession numbers of isolates and reference strains used for the ITS-based single-gene phylogenetic analysis.

Species	Strain	Host	Country	ITS
Alternaria alternata	Dcp-2	Codonopsis pilosula	China	OM334894
Alternaria alternata	Aa9L-19Qr	Quercus rubra	Poland	MN593193
Alternaria sp.	6	tobacco	China	KR920035
Alternaria infectoria	CSK1-5	Salicornia europaea	Poland	MK460778
Alternaria sp.	MBD-1164	Dalea purpurea	USA	MK595471
Alternaria sp.	G59	milk thistle	USA	KM215626
Diplodia mutila	AS7	Prunus dulcis	USA	MZ079028
Diplodia mutila	KARE592	Prunus dulcis	USA	MN166030
Botryosphaeria dothidea	BdALM10	Prunus dulcis	Spain	OQ672347
Botryosphaeria dothidea	l8-5.2	Arctostphylos glauca	USA	MW289981
Neoscytalidium novaehollandiae	CBS 122071	Mangifera indica	Australia	KF766207
Aspergillus sp.	SA-2014	soil	Egypt	KJ584852
Aspergillus sp.	BMLC4-6	glass surface	Viet Nam	MN394139
Aspergillus fumigatus	S96	Homo sapiens	Sudan	MW604211
Aspergillus fumigatus	C49N	soil	Unknown	OP237380
Aspergillus ochraceus	NRRL 398	soil	USA	EF661419
Aspergillus ochraceus	ATHUM9535	indoor air	Greece	MT990727
***Diplodia* spp.**	**KSPM 296**	**Prunus domestica**	**China**	**PX703633**
***Diplodia* spp.**	**KSPM 293**	**Prunus domestica**	**China**	**PX703635**
***Diplodia* spp.**	**KSPM 291**	**Prunus domestica**	**China**	**PX703634**
***Neoscytalidium* spp.**	**KSPM 6**	**Prunus domestica**	**China**	**PX703636**
***Neoscytalidium* spp.**	**KSPM 2**	**Prunus domestica**	**China**	**PX703637**
***Alternaria* spp.**	**KSPM 108**	**Prunus domestica**	**China**	**PX457279**
***Alternaria* spp.**	**KSPM 15**	**Prunus domestica**	**China**	**PX457287**
***Alternaria* spp.**	**KSPM 121**	**Prunus domestica**	**China**	**PX457275**
***Alternaria* spp.**	**KSPM 120**	**Prunus domestica**	**China**	**PX457276**
***Alternaria* spp.**	**KSPM 116**	**Prunus domestica**	**China**	**PX703643**
***Alternaria* spp.**	**KSPM 112**	**Prunus domestica**	**China**	**PX457277**
***Alternaria* spp.**	**KSPM 57**	**Prunus domestica**	**China**	**PX703642**
***Alternaria* spp.**	**KSPM 149**	**Prunus domestica**	**China**	**PX457269**
***Alternaria* spp.**	**KSPM 47**	**Prunus domestica**	**China**	**PX457282**
***Alternaria* spp.**	**KSPM 58**	**Prunus domestica**	**China**	**PX703641**
***Alternaria* spp.**	**KSPM 103**	**Prunus domestica**	**China**	**PX703640**
***Alternaria* spp.**	**KSPM 50**	**Prunus domestica**	**China**	**PX457281**
***Alternaria* spp.**	**KSPM 56**	**Prunus domestica**	**China**	**PX703639**
***Alternaria* spp.**	**KSPM 46**	**Prunus domestica**	**China**	**PX703638**
***Alternaria* spp.**	**KSPM 25**	**Prunus domestica**	**China**	**PX457285**
***Alternaria* spp.**	**KSPM 101**	**Prunus domestica**	**China**	**PX457280**
***Alternaria* spp.**	**KSPM 140**	**Prunus domestica**	**China**	**PX457270**
***Alternaria* spp.**	**KSPM 134**	**Prunus domestica**	**China**	**PX457274**
***Alternaria* spp.**	**KSPM 40**	**Prunus domestica**	**China**	**PX457284**
***Alternaria* spp.**	**KSPM 8**	**Prunus domestica**	**China**	**PX457289**
***Alternaria* spp.**	**KSPM 202**	**Prunus domestica**	**China**	**PX457267**
***Aspergillus* spp.**	**KSPM 35**	**Prunus domestica**	**China**	**PX703644**
***Aspergillus* spp.**	**KSPM 9**	**Prunus domestica**	**China**	**PX703645**
***Aspergillus* spp.**	**KSPM 31**	**Prunus domestica**	**China**	**PX703646**
***Aspergillus* spp.**	**KSPM 104**	**Prunus domestica**	**China**	**PX703647**
***Aspergillus* spp.**	**KSPM 106**	**Prunus domestica**	**China**	**PX703648**
***Aspergillus* spp.**	**KSPM 23**	**Prunus domestica**	**China**	**PX703649**
***Aspergillus* spp.**	**KSPM 26**	**Prunus domestica**	**China**	**PX703650**

Note:New sequences are in bold
